# Preliminary Investigation of GC–MS Profiling and Antibacterial Activities of Different Solvent Extracts From *Litchi chinensis* Sonn. Seed

**DOI:** 10.1155/sci5/7644558

**Published:** 2025-06-18

**Authors:** Samia Sharmin, Ali Ahsan Muzahid, Md. Mohibul Islam, Mst. Sarmina Yeasmin, Amit Kumar Dey, Md. Jasim Uddin, G. M. Masud Rana, Jaytirmoy Barmon, Safaet Alam, Md. Nurul Huda Bhuiyan, Nazim Uddin Ahmed

**Affiliations:** ^1^Drugs and Toxins Research Division, Bangladesh Council of Scientific and Industrial Research (BCSIR), Rajshahi Laboratories, Rajshahi 6206, Bangladesh; ^2^Oils, Fats, and Waxes Research Division, Bangladesh Council of Scientific and Industrial Research (BCSIR), Rajshahi Laboratories, Rajshahi 6206, Bangladesh; ^3^Chemistry Discipline, Khulna University, Khulna 9208, Bangladesh; ^4^Applied Botany Research Division, Bangladesh Council of Scientific and Industrial Research (BCSIR), Rajshahi Laboratories, Rajshahi 6206, Bangladesh; ^5^Institute of Food Science and Technology, Bangladesh Council of Scientific and Industrial Research (BCSIR), Dhaka 1205, Bangladesh; ^6^Fruits and Food Processing and Preservation Research Division, Bangladesh Council of Scientific and Industrial Research (BCSIR), Rajshahi Laboratories, Rajshahi 6206, Bangladesh

**Keywords:** antibacterial activity, bioactive compounds, GC–MS analysis, *Litchi chinensis*, solvent extracts

## Abstract

Traditionally different parts of *Litchi chinensis* Sonn. (Family: Sapindaceae) have been used medicinally to treat a variety of diseases, including stomach ulcers, flatulence, obesity, cough, diabetes, and hernia-like situations. This study investigates the gas chromatography–mass spectrometry (GC–MS) profiling to detect different types of phytochemicals and antibacterial activity of various solvent extracts derived from the seeds of *Litchi chinensis* Sonn. Eventually, comprehending the potential biological functions of the detected compounds is explored. GC–MS analysis revealed a diverse array of chemical compounds, with 34, 35, and 25 compounds identified in the n-hexane, n-hexane–chloroform (2:1), and methanol extracts, respectively. The major compounds identified were 2,4-bis (1,1-dimethylethyl) phenol (14.38%) in the n-hexane extract, hexadecenoic acid (13.35%) in the n-hexane–chloroform extract, and 2-(hydroxymethyl)-2-nitro-1,3-propanediol (39.16%) in the methanol extract. While most compounds exhibited biological activity, some were neutral or fatty acid derivatives. Notable bioactive compounds included bis (2-ethylhexyl) phthalate and pentadecane in the n-hexane extract, naphthalene in the n-hexane–chloroform extract, and 13-docosanamide (Z) and beta-longipinene in the methanol extract. Antibacterial activity was tested against *Listeria monocytogenes* and *Salmonella choleraesuis,* where methanolic extract showed the highest activity (ZOI-10 mm for both bacteria), followed by n-hexane extract and n-hexane–chloroform (2:1) extract, respectively. The study's findings highlight the potential of *L. chinensis* seed extracts as sources of bioactive compounds, supporting their traditional medicinal uses and suggesting possible applications in antimicrobial therapy. Future studies should focus on isolating and characterizing the key bioactive compounds and broadening the scope to test against additional pathogens and assess other biological activities, such as anticancer and anti-inflammatory effects, could further validate the medicinal uses of *L. chinensis* Sonn.

**Summary**

• Sixty-three compounds were identified by GC–MS profiling from the litchi seed extracts.
• Biologically active compounds along with some neutral or fatty acid derivatives were found.
• All extracts exhibited antibacterial activity both gram-positive and gram-negative bacteria.

## 1. Introduction

Plants, due to their natural abundance, research on medicinal plants continues to thrive, ensuring their ongoing relevance as vital sources of medicine [1]. Infectious diseases pose a significant global health challenge [2], which account for 20% of all mortality and 25% of hospital deaths annually [3]. Based on recent advancements in clinical research, it has been discovered that bioactive phytoconstituents play expedient functions against these microbial attacks [3, 4]. Although there are many commercially available antimicrobial medications, rising resistance and related mortality are requiring researchers to consistently find new antibiotics [5]. Furthermore, to lower the death rate, researchers are being compelled by rising healthcare costs and mortality rates to develop novel antimicrobials with fewer adverse effects by employing natural product chemistry [4, 6]. The study of natural products has seen a minor decline in attention due to the recent advancements in combinatorial chemistry for drug discovery and development, but nature is still considered a priceless source of enormous chemical variety and fragments that help produce new lead compounds [7, 8]. Additionally, just 6% of plants have undergone biological screening globally [5, 9]. As a result, there is a growing need for thorough research into unknown medicinal plants with potential biological activity, which will hopefully lead to the identification of numerous interesting drug candidates [10, 11]. Since the ancient period, medicinal plants have played a crucial role in ensuring human welfare. It has been estimated that 66% of plant species in the world have therapeutic qualities [3, 12]. These therapeutic plants can be used in medications or formulations to treat a variety of human disorders since they have a variety of therapeutic components [4]. Due to their natural abundance, the possibility of doing studies with these medicinal plants will not disappear quickly [13]. As a consequence, they have been vital sources of medicine for at least 60 millennia, according to fossil records [12, 14]. These days, higher plants are used to manufacture 25% of natural items, whereas 50% of all appropriate medications come from natural products and their results [5].

Although various parts of *Litchi chinensis* Sonn. (Sapindaceae) have traditionally been used in medicine to treat a range of ailments, including stomach ulcers, flatulence, obesity, cough, diabetes, and hernia-like conditions [15], scientific research has increasingly focused on evaluating the pharmacological properties of *Litchi chinensi* Sonn. and its bioactive compounds [16]. Studies have demonstrated its potential antioxidant [17], anti-inflammatory [18], antimicrobial [19], anticancer [15], and antidiabetic [20] effects, which support its traditional uses. Additionally, modern research is exploring the molecular mechanisms behind its therapeutic actions [21], paving the way for more evidence-based applications in clinical settings [22].

In plant life cycle, seeds time span is short, but the active biochemical pathways are more similar with that of animal organism because as energy source they utilize similar kind of biomolecules such as carbohydrate, fat, and protein. That is why the smaller molecules available in seeds that regulate and rectify the metabolic pathway [23] can easily be exploited for design and development of drugs for animal organisms.

One of the previous finding has shown that seven flavonoid glycosides with one new compound (litchioside D) were isolated from the seeds of lychee using repeated column chromatography and high-performance liquid chromatography (HPLC) [22]. While studies on *Litchi chinensis* Sonn. seed extracts have demonstrated various bioactive compounds with potential antimicrobial, anticancer, and glucose-lowering properties, grown in various countries, limited research has been conducted in Bangladesh on *Litchi chinensis* Sonn seed extracts. A study was conducted to evaluate the antioxidant potential of two extracts (aqueous and ethanol) to explore the relationship between phytochemicals and antioxidant activities [24], but that study did not conduct gas chromatography–mass spectrometry (GC–MS) analysis and antibacterial screening.

The goal of the current investigation was to explore the bioactive phytochemicals present in different solvent extracts of *L. chinensis* seeds and to assess their antimicrobial properties. By correlating the phytochemical profiles with potential pharmacological actions, this research seeks to uncover new insights into the therapeutic potential of *L. chinensis* and contribute to the ongoing search for effective natural antimicrobial agents.

Additionally, most studies lack comprehensive in vivo and in vitro evaluations to confirm efficacy, toxicity, and pharmacokinetics. Furthermore, research on the broader spectrum of biological activities, the potential development of these compounds into clinical applications, remains underexplored. Addressing these gaps could unlock new insights into the medicinal value of *L. chinensis* and its applications in pharmaceutical development.

## 2. Materials and Methods

### 2.1. Materials, Chemicals, and Reagents

Seeds of *Litchi chinensis*, methanol (analytical grade, purity 99.8%, Sigma-Aldrich, France), n-hexane (analytical grade, purity 97.0%, Sigma-Aldrich, France), chloroform (analytical grade, purity 98.0%, Merck, Germany), azithromycin (pharmaceutical secondary standard, purity 96.0%, Merck, Germany).

### 2.2. Plant Collection and Identification

From May 16 to 18, 2022, fresh seeds of *Litchi chinensis* Sonn. were collected from the Rajshahi city (24.3746°N and 88.6004°E) of Bangladesh. The plant specimens were identified by botanist Dr. A. N. Chowdhury, Principal Scientific Officer at Principal Scientific Officer, Bangladesh Council of Scientific and Industrial Research (BCSIR), Rajshahi Laboratories. The sample has been assigned the accession number FK-156.

### 2.3. Solvent Extraction of *Litchi chinensis*

The seeds were air-dried at room temperature for two days before being ground using a grinder (Mixture Grinder, India). Three portions (each of 300 g) of the powdered seeds were then subjected to cold extraction in three different solvent systems with increasing polarity: n-hexane < n-hexane: chloroform (2:1 ratio) < methanol. The mixtures were left to stand at room temperature for 72 h with regular manual shaking. Afterward, the extracting solvents were filtered through Whatman No. 1 filter paper, and the filtrates were concentrated using a rotary evaporator [25]. This process yielded three different extracts, which were stored at 4°C in refrigerator until further analysis.

### 2.4. Sample Preparation for GC–MS Examination

Three 50 mL Falcon tubes were labeled with serial numbers. In each tube, 350 mg of powdered *Litchi chinensis* seed was placed. The first tube received 15 mL of n-hexane, the second tube received 15 mL of a mixture of n-hexane and chloroform, and the third tube received 15 mL of methanol. The mixtures were vortexed in the tubes until they lost color. Subsequently, the tubes were heated in a water bath at 50°C for 2 h. After heating, the organic layer of each mixture was carefully extracted using a micropipette and prepared for GC–MS analysis.

### 2.5. GC–MS Analysis

The sample was analyzed using a GC–MS system (Shimadzu GCMS-QP-2020, Shimadzu, Japan) equipped with an autosampler (AOC-20s) and an autoinjector (AOC-20i) [26]. Helium gas was used as the mobile phase (carrier gas) with a flow rate of 1.72 mL/min, and the column flow rate was set at 0.75 mL/min. The column used was an SH Rxi 5MS Sill (30 m × 0.25 mm; 0.25 μm). The oven temperature was initially set to 80°C for 2 min and then increased at a rate of 5°C per min until reaching 150°C, where it was held for 5 min. The temperature was then further increased to a final temperature of 280°C, where it was maintained for 5 min.

The injector and ion source temperatures were set at 220°C and 280°C, respectively. A 5.0 μL sample was injected in splitless mode with a split ratio of 50:1. The mass spectrometer operated with an ionization potential of 70 eV, and mass spectra were recorded in the range of 45 m/z to 350 m/z over a 50-min run time. The solvent cut time was 3.00 min. Compounds were identified by comparing the mass spectra to the NIST08s, NIST08, and NIST14 libraries. The relative percent quantity of each component was determined by comparing the average peak area of each component to the total areas.

### 2.6. Identification of Chemical Constituents

The identification of bioactive compounds in various extracts of *L. chinensis* seeds was accomplished by analyzing the GC–MS retention time (RT) on the SH-Rxi 5Sill MS column and the molecular fragment m/z values. The obtained RT and m/z values from the spectra were compared using NIST08s, NIST08, and NIST14 software. The database contains approximately 60,000 recognized compound patterns. The spectra of *Litchi chinensis* seed extracts were compared to reference mass spectra of known components stored in the NIST08 and NIST14 libraries.

### 2.7. Plant Extracts Antibacterial Activity Test

The antibacterial activity of various solvent extracts of *L. chinensis* seed powder was evaluated using the disc diffusion method with slide modifications [27, 28] on nutrient agar (NA) plates. Two bacterial strains were tested in this study: *Salmonella choleraesuis* ATCC 10708, a gram-negative bacterium, and *Listeria monocytogenes* ATCC 13932, a gram-positive bacterium. The test organisms were first inoculated into nutrient broth and incubated overnight at 37°C. In this study, standard antibiotic azithromycin (15 μg/disc) used as the positive control, along with dimethyl sulfoxide (DMSO) serving as the negative control, was subsequently positioned on the NA media. The turbidity of the bacterial suspension was then adjusted to match a 0.5 McFarland standard, resulting in a final inoculum concentration of 1.5 × 10^8^ CFU/mL. After allowing the extracts to diffuse at room temperature for 30 min, the plates were incubated at 37°C for 24 h. Post incubation, the plates were examined for the presence of clear zones around the wells, indicating antibacterial activity. The diameter of these zones of inhibition (ZOIs) was measured in millimeters (mm).

### 2.8. Statistical Analysis

The SPSS software (IBM, Version 20) was used to conduct the statistical analysis, which included one-way analysis of variance (ANOVA) test (Duncan as post hoc) and independent sample *t* test (*p* < 0.05). All the experimental findings were represented as mean ± standard deviation (SD).

## 3. Results

**Figure 1:**
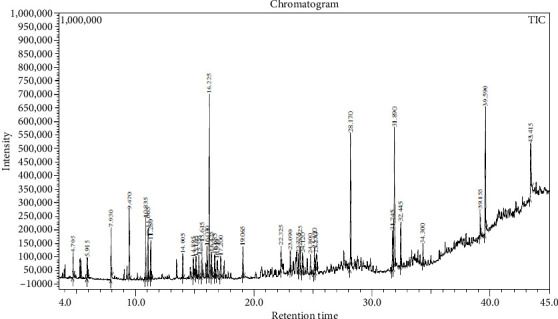
GC–MS chromatogram of n-hexane extract of *Litchi chinensis* seed.

**Figure 2:**
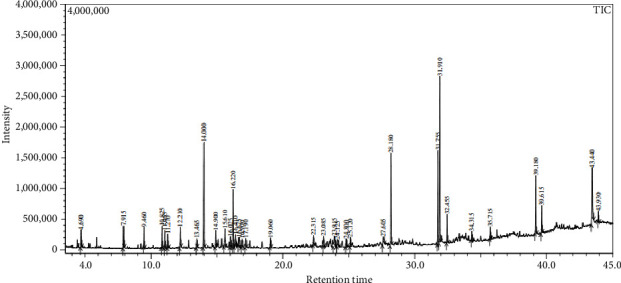
GC–MS chromatogram of n-hexane–chloroform (2:1) extract of *Litchi chinensis* seed.

**Figure 3:**
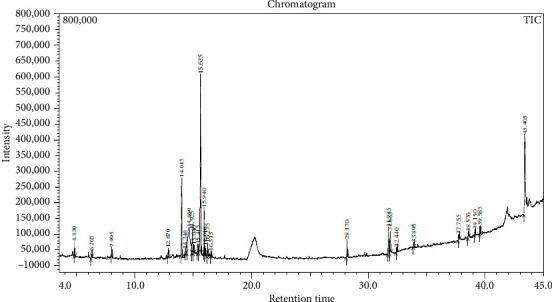
GC–MS chromatogram of methanol extract of *Litchi chinensis* seed.

Across three different extracts, a total of 34 compounds were identified in the n-hexane extract, 35 in the n-hexane–chloroform (2:1) extract, and 25 in the methanol extract. The majority of these compounds were bioactive. The chromatograms are shown in Figures 1, 2, and 3, while the chemical constituents, along with their RT, mass-to-charge ratio (m/z), and biological activity, are presented in Tables 1, 2, and 3.

However, employing three solvent systems, a total of 63 compounds were found in the powdered *L. chinensis* seed (Table 4). The main compounds found by GC–MS analysis using methanol extract were 13-docosanamide, Z (10.94%), 2-(hydroxymethyl)-2-nitro 1, 3-propanediol (39.16%), and beta-longipinene (10.02%). However, phenol, 2,4-bis (1,1-dimethylethyl) (14.38%), bis (2-ethylhexyl) phthalate (10.69%), and hexadecanoic acid methyl ester (9.75%) were the primary compounds in the n-hexane extract, where hexadecenoic acid methyl ester (13.35%), phenol, 2,4, bis(1,1-dimethylethyl) (9.61%), and 9-octadecenoic acid (8.49%) were present in n-hexane–chloroform (2:1). Six common compounds were found in all three extracts from Table 4: caryophyllene, naphthalene, benzene acetaldehyde, 9,12-octadecadienoic acid methyl ester, hexadecanoic acid methyl ester, and methyl stearate.

Other than the aforementioned six, following 16 compounds, for example, benzene 1,3-bis (1,1-dimethylethyl), 2-isopropyl-5-methyl-1-heptanol, isotridecanol, pentadecane, phenol, 2,4, bis(1,1-dimethylethyl), 2-hexyl-1-octanol, hexadecane, 1-decanol 2-hexyl-1-decanol, 2-octyl-1-dodecanol, 2-octyl-11-methyl dodecanol, heneicosane, octatriacontyl trifluoroacetate, 13-docosenoic acid, methyl ester, (Z), bis (2-ethylhexyl) phthalate, and *cis*-11-eicosenamide, were present both in the n-hexane and n-hexane–chloroform (2:1) extracts (Tables 1, 2); three compounds, alpha-caryophyllene, beta-longipinene, and methyl 9,10-methylene octadecanoate, were found in both n-hexane–chloroform (2:1) and methanol extract (Tables 2 and 3), and only 11-octadecanoic acid methyl ester was common compound detected in n-hexane and methanol extract. But the concentration of the compounds varied depending on the solvent system. Ten compounds, 2,6,11-trimethyl-dodecane, nonanal, 4,6-dimethyl dodecane, 3,7,11-trimethyl-1-dodecanol, aromandendrene, eicosane, tetratriacontyl heptafluorobutyrate, carbonic acid, decyl undecyl ester, eicosyl octyl ether, and 2,6,10,14-tetramethyl hexadecane, were available only in n-hexane extract, and another set of 10 compounds, 2,4-diethyl 1-heptanol, 2-methoxy-4-(2-propenyl)-phenol acetate, tetradecane, n-tridecane-1-ol, 1-octadecane sulfonyl chloride, 2-hexyl 1-dodecanol, 2,6,10,15-tetramethyl heptadecane, methyl ester 9-octadecanoic acid, and squalene, were detected only in n-hexane–chloroform (2:1) extract, and 15 compounds, phenylethyl alcohol, copaene, *trans*-alpha-bergamoetene, 1,3-propanediol, 2-(hydroxymethyl)-2 nitro, cis-alpha-bisabolene, spiro [4,5] dec-7-ene,1,8-dimethyl-4-(1-methyl), gamma-elemene, naphthalene 1,2,4a,5,6,8a-hydroxy-4,7-dimethylethyl, bicyclo [7.2.0]undec-4-ene, 4,11,11-trimethyl, naphthalene, 1,2,3,5,6,8a-hexahydro-4,7-dimethylethyl, 3,7-nonadien-2-ol,4,8-dimethyl, 9,12-octadecadienoic acid (Z,Z)-2,3-dihydro, 9,12-octadecadienoyl chloride, mono (2-ethylhexyl) ester, (Z,Z), 1,2-benzene dicarboxylic acid, and 13-docosenamide (Z), were present only in methanol extract.

Antibacterial activity of three different extracts of *L. chinensis* seed powder is presented in Table 5. At 10 mg/mL concentration, methanol extract showed the highest ZOI of 10 mm for test organisms *L. monocytogenes and S. choleraesuis*. At the same concentration, n-hexane extract showed 8 mm and 7 mm ZOI for *L. monocytogenes and S. choleraesuis,* respectively. In case of n-hexane–chloroform extract, ZOIs were 6 and 5 mm for *L. monocytogenes and S. choleraesuis,* respectively. On the other hand, the standard azithromycin (15 μg/disc) showed that the ZOIs for *L. monocytogenes and S. choleraesuis* are 40 and 33, respectively. The percentage differences of ZOI for all extracts from standard are shown in Table 5, indicated that all extracts offered relatively moderate antibacterial activity in comparisons with standard.

Phenolic acids, tannins, flavonoids, triterpenes, anthocyanins, and sterols are among the phytochemicals of *L. chinensis* that may contribute to its many potential biological activities, including anti-inflammatory, antiviral, antimutagenic, anticancer, antioxidant, antimicrobial, antipyretic, antiplatelet, and antihyperlipidemic effects [15].

## 4. Discussion

The findings of this study align with previous research demonstrating the presence of bioactive compounds in *Litchi chinensis* extracts, which possess antimicrobial and other therapeutic properties. However, this study extends prior work by using GC–MS profiling to provide a more detailed identification of specific compounds in different solvent extracts, such as 2,4-bis(1,1-dimethylethyl) phenol, hexadecenoic acid, and 2-(hydroxymethyl)-2-nitro-1,3-propanediol, each found in higher concentrations within specific extracts (n-hexane, n-hexane–chloroform, and methanol, respectively). Three different types of esters of fatty acids were detected among which hexadecenoic acid, methyl ester, was the most prominent. The compound has been reported to have various biological properties such as antioxidant, nematicide, hypocholesterolemic, pesticide, antiandrogenic, 5-alpha reductase, and hemolytic inhibitor activities [29]. Two other fatty acid derivatives were the methyl ester of 9,12-octadecadienoic acid, which is the most prevalent fatty acid in human nutrition and is used to treat atherosclerosis and hyperlipoidemia [30] and the methyl stearate that possess different physiological functions such as GABA aminotransferase inhibition, gastrin inhibition, and antinociception [31].

The next outstanding compound was naphthalene which is hazardous for human health. A communication pheromone, benzene acetaldehyde, also called α-toluic aldehyde, phenylacetaldehyde, α-tolualdehyde, and hyacinthin, which is used for synthesizing fragrance and polymers, as well as caryophyllene, a ring-opened isomers *β*-caryophyllene [32] which is used as cardioprotective, hepatoprotective, and immune‐modulatory in pharmaceuticals [33], were also identified. Caryophyllene elicits antimicrobial by altering membrane permeability and integrity of the target cell [34] and antitumor activity by condensing chromatin block and fragmenting DNA [35]. 1,1-Dimethylethyl-2,4-bis, the most prevalent of the 15 chemicals found in n-hexane and n-hexane–chloroform extracts, was phenol. The compound has antifungal, antimicrobial, antioxidant, and antimalarial activities [36]. It impedes quorum sensing of organism to reduce the growth rate of pathogenic fungi and bacteria [37] by inhibiting the biofilm formation [38]. The next prominent compound concurrently detected in these two extracts was bis (2-ethylhexyl) phthalates. The compound is highly antibacterial and cytotoxic [39]. Different hydrocarbon compounds such as hexadecane, benzene 1,3-bis (1,1-dimethylethyl), heneicosane, and pentadecane appeared in these two extracts are reported to have various types of medicinal properties, e.g., lipid oxidation [40], antifungal, antibacterial, antioxidant, sgar-phosphatase inhibitor, chymosin inhibitor, antibacterial, antineoplastic, and oviposition-attractant pheromone. Pentadecan's sugar-phosphatase inhibitor activity [41] could be a clue to explain the cause of death of children that were reported in Bangladesh and India. Cis-11-eicosenamide, an anti-inflammatory and antioxidant [42], was available both in n-hexane (3.333%) and n-hexane–chloroform (4.18%) extracts. Although the six alcoholic hydrocarbon viz. isotridecanol, 1-decanol, 2-octyl 2-hexyl-1 octanol, 2-isopropyl-5-methyl-1-heptanol, 1-dodecanol, 2-octyl, 1-decanol 2-hexyl as well as octatriacontyl trifluoroacetate, 13-docosenoic acid, methyl ester (Z) in negligible amount, they are reported to antimicrobial and fragrance [43], anti-inflammatory, and anticancer properties. Alpha-caryophyllene, the major compound among the common three compounds detected in methanol and n-hexane–chloroform extracts, is an 11-member monocyclic sesquiterpene and is reported to possess potent anti-inflammatory properties [44]. 11-Octadecenoic acid methyl ester was detected both in n-hexane and methanol extract which is used as an antioxidant in oil-based formulation [45]. Hexadecane, 2,6,10,14-tetramethyl which is used as biomarker; eicosane that have antifungal, antitumor, larvicidal, and cytotoxic properties [41]; and eicosyl octyl ether that is available in different seed oils [46] were more than 2% among the 11 compounds detected only in n-hexane extract. 9-Octadecenoic acid methyl ester that has anticancer and antioxidant activities [47] was the most prominent compound available only in n-hexane–chloroform extract. 1,3-Propanediol, 2-(hydroxymethyl)-2 nitro, gamma-elemene, and 13-docosanamide (Z) were the most prominent compounds available only in methanol extract. The compound 1,3-propanediol, 2-(hydroxymethyl)-2 nitro has reportedly been shown to be microbicidal and is used as a bacterial growth inhibitor and disinfectants [48]. The second highest compound was 13-docosanamide Z (10.94%) in methanol extract. It exerts antinociceptive activities [49] with a possible mechanism of having its structural analogy with docosahexaenoic acid (DHA) which is a very important component of nerve of the brain [50]. An interesting future of all group of bioactive compounds detected is that they are not only diversified in biological activity but also some of them were opposing in their biological function. For example, naphthalene is a carcinogenic compound whereas eicosane is an antitumor one and octatriacontyl trifluoroacetate is an insecticidal, but heneicosane is oviposition-attractant pheromone chemical and bis (2-ethylhexyl) phthalate increases cellular proliferation [51]. Other anticancer compounds were 13-docosenoic acid, methyl ester (Z) [52]. Squalene, precursor of all plant and animal sterols, has also biological activities such as antibacterial, antioxidant, antitumor, cancer preventive, immunostimulant, and pesticide [31]. 9,12-Octadecadienoic acid, a ω-6 fatty acid, is not only antioxidative and hypolipidemic [30] but also an enhancer of antibacterial and antifungal activities. It exerts the later effect by increasing the permeability of the cell membrane. These results reveal the variation in bioactive content depending on the extraction solvent, a detail that previous studies may not have examined as thoroughly.

The antibacterial activity of methanolic extract showed high antibacterial activity (ZOI, 10 mm) against *L. monocytogenes* and *S. choleraesuis* followed by n-hexane extract and n-hexane–chloroform extract, respectively (Table 5). Methanolic extract showed high antibacterial activity (ZOI, 10 mm) against *L. monocytogenes* and *S. choleraesuis* followed by n-hexane extract and n-hexane–chloroform extract, respectively (Table 5). The result indicates that extractives of polar solvent system have notable, nonpolar solvent system has medium antimicrobial activity and mixed solvent system has lowest antimicrobial activity in comparison among them. The presence of highest concentration of 2-(hydroxymethyl)-2 nitro 1,3-propanediol (39.16%) in the methanolic extract elucidates its highest antibacterial activity against tested bacteria. The antibacterial activity of lychee probably attributed to a variety of mechanisms, as supported by the body of existing literature and the structural characteristics of the main compounds found within them. The primary mode of action involves disruption of the bacterial cell membrane, leading to increased permeability, leakage of intracellular contents, and eventual cell lysis. Additionally, certain bioactive constituents, such as terpenes and alcohols, are known to inhibit key enzymatic processes essential for bacterial survival and metabolism [53, 54].

In terms of antimicrobial activity, this study's results align with previous findings that methanolic extract *L. chinensis* Sonn. seed exhibits effectiveness against certain pathogens: *Klebsiella pneumoniae* with a ZOI of 15 mm, *Pseudomonas aeruginosa* (ZOI-12 mm), and *Staphylococcus aureus* (ZOI-9 mm) [55]. Another study report showed by the disc diffusion method, *L. chinensis* of aqueous extract was found to have moderate antibacterial activity against gram-positive bacteria such as *Staphylococcus aureus, Streptococcus pyogenes, and Bacillus subtillis*, as well as Gram-negative bacteria such as *Escherichia coli and Pseudomonas aeruginosa* [56]. These findings are consistent with our research indicating that the methanol extract demonstrated notable activity against *Listeria monocytogenes* and *Salmonella choleraesuis*. This could be due to differences in extraction methods, solvent choice, or plant material, suggesting that extraction conditions significantly influence the antibacterial efficacy. Overall, while this study supports existing knowledge of *L. chinensis*'s medicinal properties, it offers new insights into solvent-dependent bioactivity and specific bioactive profiles, encouraging more targeted exploration of extraction techniques and compound-specific activities in future research.

## 5. Conclusions

In this study, GC–MS analysis of three different extracts n-hexane, n-hexane–chloroform (2:1), and methanol revealed the presence of 63 bioactive compounds. These compounds, which vary in structure and function, suggest their involvement in cellular differentiation, proliferation, and protection against oxidative damage and pathogenic attacks. Antimicrobial potentiality tested against certain pathogens; *Listeria monocytogenes* and *Salmonella choleraesuis* confirmed their antimicrobial activity. Furthermore, the detected compounds align with previously reported glucose-lowering effects, further validating the therapeutic potential of *L. chinensis* extracts. The diversity of these bioactive molecules supports the possible medicinal uses of the *Litchi chinensis* Sonn. seed. Overall, the findings highlight the therapeutic potential of *L. chinensis* extracts and justify further exploration of its bioactive compounds for their medicinal applications. Finally, we can conclude that methanolic extract shows the more prominent compounds from other two extract which help to express potential activity against bacteria, so this extract is relatively best from other two. Further studies are required to isolate compounds from the *L. chinensis* extracts, purification and structural elucidation bioactive compounds, along with in-depth pharmacological evaluations, to fully understand their mechanisms of action and potential applications in drug development. The results of this study offer significant understanding of the bioactive potential of L. chinensis extracts, paving the way for further research on compound isolation, purification, and their formulation for use in pharmaceuticals.

## Figures and Tables

**Table 1:** Phytochemical constituents identified in n-hexane extract of *L. chinensis* seed powder by GC–MS analysis.

No.	Name of compound	RT	m/z	Biological activity
1	Benzene acetaldehyde	4.71	91.00	Antibacterial activity
2	Nonanal	5.92	57.00	Antimicrobial activity
3	Naphthalene	7.93	128.00	Antimicrobial, antiseptic, carcinogenic
4	Benzene 1,3-bis (1,1-dimethylethyl)	9.48	175.00	Lipid oxidation
5	1-Decanol, 2-hexyl-	10.84	69.00	Commercially used
6	Isotridecanol	11.07	69.00	Antimicrobial
7	2-Isopropyl-5-methyl-1-heptanol	11.29	69.00	Antimicrobial
8	Caryophyllene	14.01	91.00	Cardioprotective, hepatoprotective, and immune‐modulatory activity
9	Dodecane, 2,6,11-trimethyl	14.90	71.00	Antifungal, antibacterial activities
10	Hexadecane	15.11	57.00	Antifungal, antibacterial, antioxidant
11	Dodecane 4,6-dimethyl	15.34	71.00	—
12	Aromadendrene	15.62	93.00	Antimicrobial, antifungal
13	Pentadecane	16.04	57.00	Suger-phosphatase inhibitor, chymosin inhibitor, antibacteria
14	Phenol, 2,4, bis (1,1-dimethylethyl)	16.23	191.00	Antifungal, antimicrobial, antioxidant, antimalarial activities
15	1-Dodecanol,3,7,11-trimethyl	16.42	69.00	—
16	11-Methyl dodecanol	16.69	69.00	—
17	1-Decanol, 2-octyl	16.92	57.00	—
18	2-Hexyl-1-octanol	17.20	69.00	Antihelminthic
19	Eicosane	22.33	57.00	Antifungal, antitumor, larvicidal
20	Tetratriacontyl heptafluorobutyrate	23.10	69.00	Antimicrobial property
21	Carbonic acid, decyl undecyl ester	23.78	57.00	—
22	Eicosyl octyl ether	23.93	57.00	—
23	1-Dodecanol, 2-octyl	24.13	69.00	Emollients, perfuming agents, cosmetics
24	Octatriacontyl trifluoroacetate	24.80	69.00	Insecticidal
25	Heneicosane	25.12	57.00	Antineoplastic, oviposition-attractant
26	Hexadecane, 2,6,10,14 tetramethyl	25.28	57.00	Biomarkers in petroleum studies
27	Hexadecanoic acid, methyl ester	28.17	74.00	Nematicide, pesticide, hemolytic, 5-alpha reductase inhibitor activities
28	9,12-Octadecadienoic acid	31.75	67.00	Antibacterial, antifungal and antioxidative, hypolipidemic
29	11-Octadecanoic acid	31.90	55.00	Antioxidants
30	Methyl stearate	32.45	74.00	GABA aminotransferase inhibitor, gastrin inhibitor, antinociceptive
31	1-Decanol 2-hexyl	34.30	69.00	Commercially used
32	13-Docosenoic acid, methyl ester, (Z)-	39.16	55.00	Anticancer
33	Bis (2-ethylhexyl) phthalate	39.60	149.00	Antimicrobial and cytotoxic activity
34	*Cis*-11-eicosenamide	43.42	59.00	Antioxidant and anti-inflammatory effects

**Table 2:** Phytochemical constituents identified by GC–MS analysis in n-hexane–chloroform (2:1) extract of *L. chinensis* seed powder.

No.	Name of compound	RT	m/z	Biological activity
1	Benzene acetaldehyde	4.70	91.00	Antibacterial activity
2	Naphthalene	7.92	128.00	Antimicrobial, antiseptic, carcinogenic
3	1,3-Bis (1,1-dimethylethyl) benzene	9.46	175.00	Lipid oxidation
4	2-Isopropyl-5-methyl-1-heptanol	10.83	57.00	Antimicrobial
5	Isotridecanol	11.05	69.00	Antimicrobial
6	2,4-Diethyl, 1-heptanol	11.27	69.00	—
7	2-Methoxy-4-(2-propenyl)-phenol, acetate	12.21	164.00	Antiinfective-agents, antioxidants
8	Tetradecane	13.46	57.00	Antimicrobial
9	Caryophyllene	14.00	93.00	Cardioprotective, hepatoprotective, and immune‐modulatory activity
10	Alpha-caryophyllene	14.91	93.00	Anti-inflammatory
11	Beta-longipinene	15.61	91.00	Antibiofilm activity
12	Pentadecane	16.02	57.00	Sugar-phosphatase inhibitor, chymosin inhibitor, antibacterial
13	2,4, Bis (1,1-dimethylethyl), phenol	16.22	191.00	Antifungal, antimicrobial, antioxidant, antimalarial activities
14	11-Methyldodecanol	16.41	69.00	Antimicrobial
15	n-Tridecane-1-ol	16.68	69.00	Antibacterial activity
16	1-Octadecane sulfonyl chloride	16.90	57.00	—
17	2-Hexyl-1-octanol	17.19	69.00	Antihelminthic
18	Hexadecane	19.06	57.00	Antifungal, antibacterial, antioxidant
19	Heptadecane	22.32	57.00	Antioxidant
20	2-Hexyl, 1-decanol	23.08	69.00	Commercially used
21	2-Hexyl, 1-dodecanol	23.91	69.00	—
22	2-Octyl, 1-decanol	24.13	57.00	—
23	2-Octyl, 1-dodecanol	24.80	69.00	Emollients, perfuming agents, cosmetics
24	Heneicosane	25.12	57.00	Antineoplastic, oviposition-attractant pheromone
25	2,6,10,15-Tetramethyl, heptadecane	27.60	57.00	Sex hormone in algae
26	Hexadecenoic acid	28.18	74.00	Nematicide, pesticide, hemolytic, 5-alpha reductase inhibitor activities
27	9,12-Octadecadienoic acid	31.75	67.00	Antibacterial, antifungal, and antioxidative, hypolipidemic
28	9-Octadecanoic acid	31.91	55.00	Antihypertensive increases HDL and decrease LDL
29	Methyl stearate	32.46	74.00	GABA aminotransferase inhibitor, gastrin inhibitor, anthelmintic antinociceptive
30	Octatriacontyl trifluoroacetate	34.32	69.00	Insecticidal
31	Methyl 9,10- methylene- octadecanoate	35.72	55.00	—
32	13-docosenoic acid	39.18	55.00	Anticancer
33	Bis (2-ethylhexyl) phthalate	39.62	149.00	Antimicrobial and cytotoxic activity
34	*Cis*-11-eicosenamide	43.44	59.00	Antioxidant and anti-inflammatory effects
35	Squalene	43.93	69.00	Antibacterial, antioxidant, antitumor, cancer preventive, immunostimulant, pesticide

**Table 3:** Phytochemical constituents identified in methanol extract of *L. chinensis* seed powder by GC–MS analysis.

No.	Name of compound	RT	m/z	Biological activity
1	Benzene acetaldehyde	4.83	91.00	Antibacterial activity
2	Phenylethyl alcohol	6.27	91.00	Preservatives, antimicrobial
3	Naphthalene	7.99	128.00	Antimicrobial antiseptic
4	Copaene	12.87	105.00	Carcinogenic antioxidant and antiproliferative
5	Caryophyllene	14.01	93.00	Cardioprotective, hepatoprotective
6	*Trans*-alpha-bergamotene	14.33	93.00	Immune‐modulatory activity antimicrobial
7	2-(Hydroxymethyl)-2 nitro, 1,3-propanediol	14.66	57.00	Antimicrobial
8	Alpha-caryophyllene	14.93	93.00	Anti-inflammatory
9	*Cis*-alpha-bisabolene	15.04	93.00	Antibacterial effects
10	1,8-Dimethyl-4-(1-methyl), spiro [4,5] dec-7-ene	15.42	119.00	Antifungal activity
11	Beta-longipinene	15.62	93.00	Antibiofilm activity
12	Gamma-elemene	15.94	121.00	Antitumor activity
13	1,2,4a,5,6,8a-Hydroxy-4,7-dimethylethyl, naphthalene	16.01	105.00	—
14	4,11,11-Trimethyl bicyclo [7.2.0] undec-4-ene	16.24	93.00	—
15	1,2,3,5,6,8a-Hexahydro-4,7-dimethylethyl-naphthalene	16.52	119.00	—
16	Hexadecanoic acid	28.17	74.00	5-Alpha reductase inhibitor
17	9,12-Octadecadienoic acid	31.74	67.00	Antibacterial, antifungal, and antioxidative, hypolipidemic
18	11-Octadecanoic acid	31.88	55.00	Antioxidants
19	Methyl stearate	32.44	74.00	GABA aminotransferase inhibitor
20	4,8-Dimethyl, 3,7-nonadien-2-ol	33.90	69.00	
21	2,3-Dihydro, 9,12-octadecadienoic acid, ZZ	37.76	67.00	—
22	9,12-Octadecadienoyl chloride, ZZ	38.57	67.00	Anticancer, thyroid inhibitor
23	Methyl 9,10-methylene octadecanoate	39.15	55.00	—
24	1,2-Benzene dicarboxylic acid, mono (2-ethylhexyl) ester	39.58	149.00	Antimicrobial activity, antifungal
25	13-Docosanamide, Z	43.41	59.00	Antimicrobial, antinociceptive

**Table 4:** The relative concentration of chemical composition present in n-hexane, n-hexane–chloroform, and methanol solvent extracts of *L. sinensis* seed powder.

No.	Compounds name	Structure	Formula	Relative concentration (%)		
				n-Hexane	n-Hexane–chloroform (2:1)	Methanol
1.	Benzene acetaldehyde	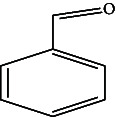	C_8_H_10_O	3.36 ± 0.016^b^	3.42 ± 0.005^b^	4.75 ± 0.012^a^
2.	Nonanal	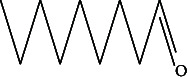	C_9_H_18_O	0.936 ± 0.009	ND	ND
3.	Phenylethyl alcohol	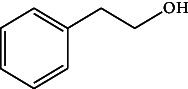	C_8_H_10_O	ND	ND	2.76 ± 0.008
4.	Naphthalene	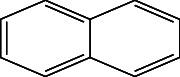	C_10_H_8_	7.24 ± 0.008^a^	5.53 ± 0.008^b^	4.20 ± 0.012^c^
5.	Benzene 1,3-bis (1,1-dimethylethyl)	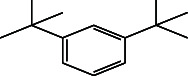	C_14_H_22_	4.866 ± 0.012^a^	2.97 ± 0.012^b^	ND
6.	1-Decanol, 2-hexyl-	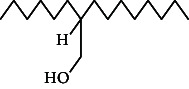	C_16_H_34_O	2.29 ± 0.043	ND	ND
7.	Isotridecanol	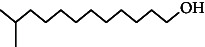	C_13_H_28_O	2.34 ± 0.008^a^	1.69 ± 0.012^b^	ND
8.	2,4-diethyl, 1-heptanol	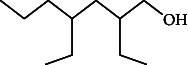	C_11_H_24_O	ND	1.10 ± 0.017	ND
9.	2-Isopropyl-5-methyl-1-heptanol	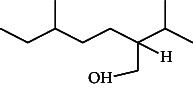	C_11_H_24_O	1.536 ± 0.009^a^	1.70 ± 0.016^a^	ND
10.	2-Methoxy-4-(2-propenyl)-phenol, acetate	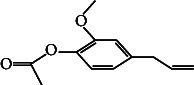	C_12_H_14_O	ND	1.78 ± 0.008	ND
11.	Copaene	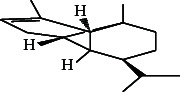	C_15_H_24_	ND	ND	1.15 ± 0.008
12.	Tetradecane	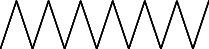	C_14_H_30_	ND	1.70 ± 0.012	
13.	Caryophyllene	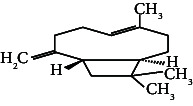	C_15_H_24_	0.503 ± 0.012^c^	4.42 ± 0.012^a^	4.59 ± 0.012^a^
14.	*Trans*-alpha-bergamotene	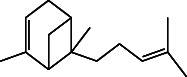	C_15_H_24_	ND	ND	0.17 ± 0.005
15.	2-(Hydroxymethyl)-2 nitro, 1,3-propanediol	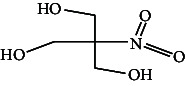	C_4_H_9_NO_5_	ND	ND	39.16 ± 0.012
16.	Propanediol	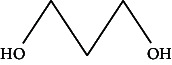	C_3_H_8_O_2_	1.11 ± 0.008	ND	ND
17.	Alpha-caryophyllene	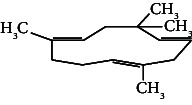	C_15_H_24_	ND	1.71 ± 0.021^b^	3.68 ± 0.008^a^
18.	*Cis*-alpha-bisabolene	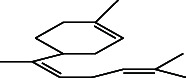	C_15_H_24_	ND	ND	0.62 ± 0.012
19.	Heptadecane, 2,6,10,15-tetramethyl-	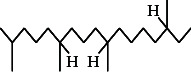	C_21_H_44_	1.4 ± 0.008	ND	ND
20.	Dodecane 4,6-dimethyl	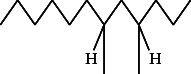	C_14_H_30_	1.19 ± 0.016	ND	ND
21.	Spiro [4,5] dec-7-ene 1,8-dimethyl-4-(1-methylethyl)	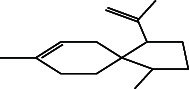	C_15_H_24_	ND	ND	0.72 ± 0.008
22.	Beta-longipinene	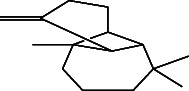	C_15_H_24_	ND	0.75 ± 0.012^b^	10.02 ± 0.012^a^
23.	Aromadendrene	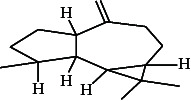	C_15_H_24_	0.75 ± 0.008	ND	ND
24.	Gamma-elemene	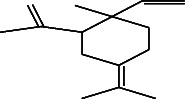	C_15_H_24_	ND	ND	4.03 ± 0.016
25.	1,2,4a,5,6,8a-Hydroxy-4,7-dimethylethyl, naphthalene	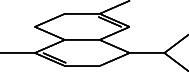	C_15_H_24_	ND	ND	0.75 ± 0.012
26.	Pentadecane	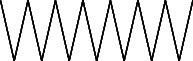	C_15_H_32_	2.326 ± 0.024^a^	1.62 ± 0.012^b^	ND
27.	Phenol, 2,4, bis (1,1-dimethylethyl)	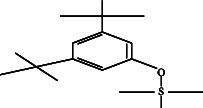	C_14_H_22_O	14.384 ± 0.014^a^	9.61 ± 0.012^b^	ND
28.	4,11,11-Trimethyl bicyclo [7.2.0] undec-4-ene	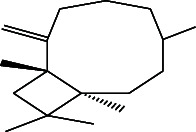	C_15_H_24_	ND	ND	0.77 ± 0.017
29.	1-Dodecanol, 3,7,11-trimethyl	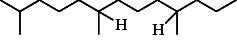	C_15_H_32_O	1.09 ± 0.008	ND	ND
30.	1,2,3,5,6,8a-Hexahydro-4,7-dimethylethyl-naphthalene	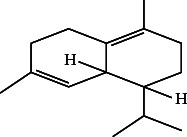	C_15_H_24_	ND	ND	0.52 ± 0.008
31.	n-Tridecane-1-ol	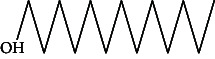	C_13_H_28_	ND	1.38 ± 0.012	ND
32.	11-Methyl dodecanol	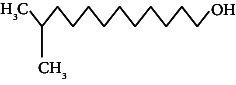	C_13_H_28_O	1.28 ± 0.008^a^	0.97 ± 0.008^a^	ND
33.	1-Octadecane sulfonyl chloride	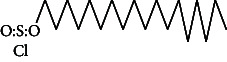	C_18_H_37_ClO_2_S	ND	1.06 ± 0.005	ND
34.	2-Octyl, 1-decanol	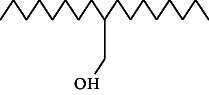	C_18_H_38_O	1.3 ± 0.024^a^	1.22 ± 0.017^a^	ND
35.	2-Hexyl-1-octanol	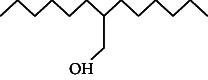	C_14_H_30_O	1.246 ± 0.012^a^	1.120 ± 0.008^a^	ND
36.	Hexadecane	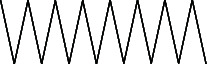	C_16_H_34_	3.48 ± 0.016^a^	2.180 ± 0.012^b^	ND
37.	Heptadecane	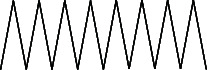	C_17_H_36_	ND	1.93 ± 0.012	ND
38.	Eicosane	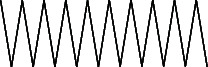	C_20_H_42_	2.24 ± 0.008	ND	ND
39.	Tetratriacontyl heptafluorobutyrate	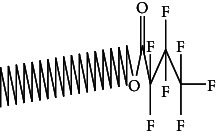	C_38_H_69_F_7_O_2_	1.23 ± 0.008	ND	ND
40.	2-Hexyl-1-dodecanol	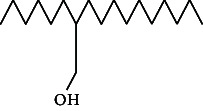	C_18_H_38_O	ND	2.03 ± 0.017	ND
41.	Carbonic acid, decyl undecyl ester	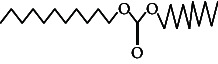	C_22_H_44_O_3_	0.763 ± 0.004	ND	ND
42.	Eicosyl octyl ether	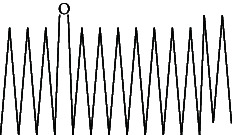	C_28_H_58_O	2.34 ± 0.008	ND	ND
43.	2-Octyl, 1-dodecanol	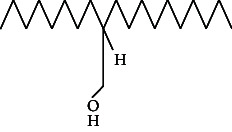	C_20_H_42_O	1.686 ± 0.009^a^	0.80 ± 0.012^b^	ND
44.	Octatriacontyl trifluoroacetate	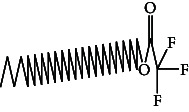	C_40_H_77_F_3_O_2_	0.963 ± 0.004^a^	0.93 ± 0.012^a^	ND
45.	Heneicosane	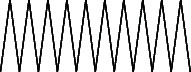	C_21_H_42_	2.093 ± 0.012^a^	1.47 ± 0.008^b^	ND
46.	Hexadecane, 2,6,10,14-tetramethyl	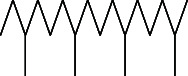	C_20_H_42_	3.033 ± 0.012	ND	ND
47.	2,6,10,15-Tetramethyl, heptadecane	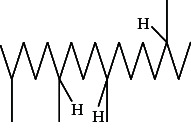	C_21_H_44_	ND	1.03 ± 0.012	ND
48.	Hexadecanoic acid, methyl ester	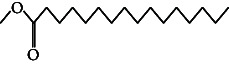	C_17_H_34_O_2_	9.753 ± 0.016^b^	13.371 ± 0.012^a^	3.16 ± 0.012^c^
49.	9,12-Octadecadienoic acid	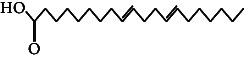	C_18_H_32_O_2_	1.196 ± 0.016^c^	5.50 ± 0.021^a^	1.90 ± 0.017^b^
50.	11-Octadecanoic acid	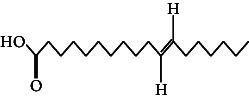	C_18_H_34_O_2_	3.673 ± 0.012^a^	ND	1.57 ± 0.008^b^
51.	9-Octadecenoic acid	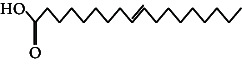	C_18_H_34_O_2_	ND	8.49 ± 0.0008	ND
52.	Methyl stearate	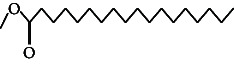	C_19_H_38_O_2_	2.79 ± 0.008^b^	3.50 ± 0.012^a^	0.56 ± 0.005^c^
53.	4,8-Dimethyl, 3,7-nonadien-2-ol	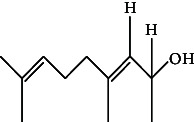	C_11_H_20_O	ND	ND	0.68 ± 0.012
54.	2-Hexyl 1-decanol	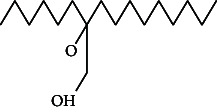	C_16_H_34_O	0.796 ± 0.004^b^	1.15 ± 0.012^a^	ND
55.	Methyl 9,10-methylene-octadecanoate	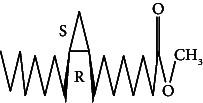	C_20_H_38_O	ND	0.63 ± 0.012^a^	0.46 ± 0.012^a^
56.	2,3-Dihydro, 9,12-octadecadienoic acid, ZZ	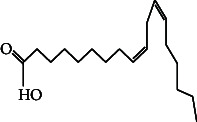	C_18_H_32_O	ND	ND	0.48 ± 0.008
57.	9,12-Octadecadienoyl chloride, ZZ	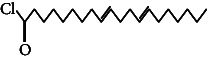	C_18_H_31_ClO	ND	ND	0.53 ± 0.012
58.	13-Docosenoic acid, methyl ester, (Z)-	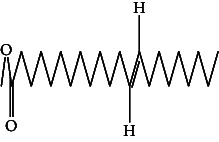	C_23_H_44_O_2_	0.79 ± 0.008^b^	2.88 ± 0.008^a^	ND
59.	1,2-Benzene dicarboxylic acid, mono (2-ethylhexyl) ester	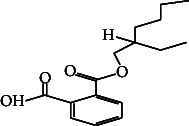	C_16_H_22_O_4_	ND	ND	1.81 ± 0.008
60.	Bis (2-ethylhexyl) phthalate	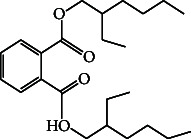	C_24_H_38_O_4_	10.69 ± 0.008^a^	4.45 ± 0.012^b^	ND
61.	*Cis*-11-eicosenamide	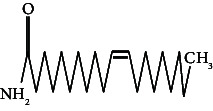	C_20_H_39_NO	3.333 ± 0.012^b^	4.18 ± 0.008^a^	ND
62.	13-Docosanamide, Z	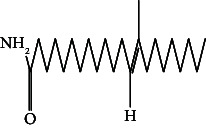	C_22_H_43_NO	ND	ND	10.94 ± 0.012
63.	Squalene	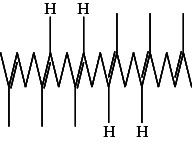	C_30_H_50_	ND	1.71 ± 0.008	ND

*Note:* Relative concentration was calculated from the percentage (%) of peak area. Values (mean ± SD) are average of three samples (*n* = 3). Different superscript letters with in the same row indicate significant difference of means (*p* < 0.05). Here, independent sample *t* test was conducted for two variables, and one-way analysis of variance (ANOVA) (Duncan test as post hoc) was for three variables.

Abbreviation: ND = not detected.

**Table 5:** Antibacterial activity of different solvent extracts of *Litchi chinensis* seed powder against microorganism at 10 mg/mL concentration.

Test organism	Zone of inhibition (ZOI) in mm						
	n-Hexane		n-Hexane–chloroform (2:1)		Methanol		Standard antibiotic (azithromycin 15 μg/disc)
	Extract ZOI	Percentage difference from standard	Extract ZOI	Percentage difference from standard	Extract ZOI	Percentage difference from standard	
*L. monocytogenes* (gram +ve)	8.0 ± 0.4^c^	80%	6.0 ± 1.1^d^	85%	10.0 ± 0.7^b^	75%	40.0 ± 1.5^a^
*S. choleraesuis* (gram −ve)	7.0 ± 1.2^c^	78.79%	5.0 ± 0.6^d^	84.85%	10.0 ± 1.3^b^	69.70%	33.0 ± 0.9^a^

*Note:* For expressing zone of inhibition (ZOI), values (mean ± SD) are analyzed individually in triplicate for each type of microorganisms. Different superscript letters within the same row indicate significant (one-way ANOVA and Duncan test as post hoc, *p* < 0.05) difference of means.

## Data Availability

The data that support the findings of this study are available from the corresponding author upon reasonable request.

## Nomenclature

ANOVA: Analysis of variance
ATCC: American Type Culture Collection
CFU: Colony forming unit
GC–MS: Gas chromatography–mass spectrometry
NIST: National Institute of Standards and Technology
SPSS: Statistical Product and Service Solutions
ZOI: Zone of inhibition

## References

[1] Newman D. J., Cragg G. M. (2016). Natural Products as Sources of New Drugs From 1981 to 2014. *Journal of Natural Products*.

[2] Bloom D. E., Cadarette D. (2019). Infectious Disease Threats in the Twenty-First Century: Strengthening the Global Response. *Frontiers in Immunology*.

[3] Alam S., Emon N. U., Shahriar S. (2020). Pharmacological and Computer-Aided Studies Provide New Insights Into Millettia Peguensis Ali (Fabaceae). *Saudi Pharmaceutical Journal*.

[4] Alam S., Rashid M. A., Sarker M. M. R. (2021). Antidiarrheal, Antimicrobial and Antioxidant Potentials of Methanol Extract of Colocasia Gigantea Hook. F. Leaves: Evidenced From In Vivo and In Vitro Studies Along With Computer-Aided Approaches. *BMC Complement Med Ther*.

[5] Kabir F., Jaman A. U., Rumpa R. A. (2021). In Vitro And In Vivo Investigations Provide New Insights Into Bioactivities of Blumea Clarkei Hook. F. Leaves. *Bangladesh Pharmaceutical Journal*.

[6] Rudra S., Sawon M., Emon N. (2020). Biological Investigations of the Methanol Extract of Tetrastigma Leucostaphylum (Dennst.) Alston Ex Mabb. (Vitaceae): In Vivo and In Vitro Approach. *Journal of Advanced Biotechnology and Experimental Therapeutics*.

[7] Alam S., Sarker M. M. R., Sultana T. N. (2022). Antidiabetic Phytochemicals From Medicinal Plants: Prospective Candidates for New Drug Discovery and Development. *Frontiers in Endocrinology*.

[8] Emon N. U., Alam S., Rudra S., Chowdhury S., Rajbangshi J. C., Ganguly A. (2020). Evaluation of Pharmacological Potentials of the Aerial Part of *Achyranthes aspera* L.: In Vivo, In Vitro and In Silico Approaches. *Advances in Traditional Medicine*.

[9] Islam M. M., Alam R., Chung H.-J. (2022). Chemical, Pharmacological and Computerized Molecular Analysis of Stem’s Extracts of *Bauhinia scandens* L. Provide Insights Into the Management of Diarrheal and Microbial Infections. *Nutrients*.

[10] Chakrabarty N., Chung H.-J., Alam R. (2022). Chemico-Pharmacological Screening of the Methanol Extract of Gynura Nepalensis DC Deciphered Promising Antioxidant and Hepatoprotective Potentials: Evidenced From In Vitro, In Vivo, and Computer-Aided Studies. *Molecules (Basel)*.

[11] Sultana N., Chung H.-J., Emon N. U. (2022). Biological Functions of Dillenia Pentagyna Roxb. Against Pain, Inflammation, Fever, Diarrhea, and Thrombosis: Evidenced From In Vitro, In Vivo, and Molecular Docking Study. *Frontiers in Nutrition*.

[12] Asad S., Kabir F., Alam S. (2022). In Vitro Analysis Provides New Insights Into the Pharmacological Actions of Methanol Extract of Seeds of *Tamarindus indica* L. And its Kupchan Fractions. *Bangladesh Pharmaceutical Journal*.

[13] Emon N. U., Rudra S., Alam S. (2021). Chemical, Biological and Protein-Receptor Binding Profiling of *Bauhinia scandens* L. Stems Provide New Insights Into the Management of Pain, Inflammation, Pyrexia and Thrombosis. *Biomedicine & Pharmacotherapy*.

[14] Chowdhury M. N. R., Alif Y. A., Alam S. (2022). Theoretical Effectiveness of Steam Inhalation Against SARS-CoV-2 Infection: Updates on Clinical Trials, Mechanism of Actions, and Traditional Approaches. *Heliyon*.

[15] Ibrahim S. R. M., Mohamed G. A. (2015). Litchi Chinensis: Medicinal Uses, Phytochemistry, and Pharmacology. *Journal of Ethnopharmacology*.

[16] Yao P., Gao Y., Simal-Gandara J. (2021). Litchi (Litchi Chinensis Sonn.): A Comprehensive Review of Phytochemistry, Medicinal Properties, and Product Development. *Food & Function*.

[17] Paliga M., Novello Z., Dallago R. M. (2017). Extraction, Chemical Characterization and Antioxidant Activity of Litchi Chinensis Sonn. And Avena Sativa L. Seeds Extracts Obtained From Pressurized N-Butane. *Journal of Food Science & Technology*.

[18] Chauhan S., Kaur N., Kishore L., Singh R. (2014). Pharmacological Evaluation of Anti-Inflammatory and Analgesic Potential of Litchi Chinensis Gaertn.(sonn.). *Group*.

[19] Kilari E. K., Putta S. (2016). Biological and Phytopharmacological Descriptions of Litchi Chinensis. *Pharmacognosy Reviews*.

[20] Chauhan S., Gupta S., Yasmin S., Saini M. (2021). Antihyperglycemic and Antioxidant Potential of Plant Extract of Litchi Chinensis and glycine Max. *International Journal of Nutrition, Pharmacology, Neurological Diseases*.

[21] Punia S., Kumar M. (2021). Litchi (Litchi Chinenis) Seed: Nutritional Profile, Bioactivities, and Its Industrial Applications. *Trends in Food Science & Technology*.

[22] Xu X., Xie H., Hao J., Jiang Y., Wei X. (2011). Flavonoid Glycosides From the Seeds of Litchi Chinensis. *Journal of Agricultural and Food Chemistry*.

[23] Ventura L., Donà M., Macovei A. (2012). Understanding the Molecular Pathways Associated With Seed Vigor. *Plant Physiology and Biochemistry*.

[24] Aktar M. N., Islam M. M., Raza M. S. (2022). Phytochemical Characteristics and Antioxidant Potential of Litchi Chinensis Sonn. Seeds of Bangladesh. *Bangladesh Journal of Agriculture*.

[25] Afroz Shoily M. S., Islam M. E., Rasel N. M. (2025). Unveiling the Biological Activities of *Heliotropium indicum* L. Plant Extracts: Anti-Inflammatory Activities, GC–MS Analysis, and In-Silico Molecular Docking. *Scientific Reports*.

[26] Khatun M. H., Sami S. A., Mim F. S. (2025). Unveiling Pharmacological Promise of Mangifera Indica (Haribhanga) Peel Extract: Exploring an Untapped Cultivar Through Biochemical and Computational Approaches. *Scientific*.

[27] Gangegoda S., Abeywardhana S., Sigera S., Nirmani A., Peiris D. C. (2024). Antioxidant and Antimicrobial Properties of Codium Fragile (Suringar) Methanol Extract: Insights From Molecular Docking Analysis. *Algal Research*.

[28] Rana G. M. M., Uddin M. J., Barmon J. (2025). Assessment of the Essential Oil Extracted From Citrus Aurantifolia Leaves Using Solvent-Free Microwave Extraction Technique. *Food Chemistry Advances*.

[29] Oni J. O., Akomaye F. A., Markson A.-A. A., Egwu A. C. (2020). GC-MS Analysis of Bioactive Compounds in Some Wild-Edible Mushrooms From Calabar, Southern Nigeria. *European Journal of Biology and Biotechnology*.

[30] Hagr T. E., Ali K. S., Satti A. A. E., Omer S. A. (2018). GC-MS Analysis, Phytochemical, and Antimicrobial Activity of Sudanese Nigella Sativa (L) Oil. *European Journal of Biomedical and Pharmaceutical Sciences*.

[31] Adnan M., Nazim Uddin Chy M., Mostafa Kamal A. T. M. (2019). Investigation of the Biological Activities and Characterization of Bioactive Constituents of Ophiorrhiza Rugosa Var. Prostrata (D. Don) & Mondal Leaves Through In Vivo, In Vitro, and In Silico Approaches. *Molecules (Basel)*.

[32] Hartsel J. A., Eades J., Hickory B. (2016). *Chapter 53. Cannabis Sativa and Hemp*.

[33] Machado C., Islam M. T., Ali E. S. (2018). A Systematic Review on the Neuroprotective Perspectives of Beta—Caryophyllene. *Phytotherapy Research*.

[34] Moo C., Yang S., Osman M., Yuswan M. H. (2020). Antibacterial Activity and Mode of Action of β-Caryophyllene on Bacillus Cereus. *Polish Journal of Microbiology*.

[35] Dahham S. S., Tabana Y. M., Iqbal M. A. (2015). The Anticancer, Antioxidant and Antimicrobial Properties of the Sesquiterpene β-Caryophyllene From the Essential Oil of Aquilaria Crassna. *Molecules (Basel)*.

[36] Elaiyaraja A., Chandramohan G. (2016). Comparative Phytochemical Profile of *Indoneesiella echioides* (L.) Nees Leaves Using GC-MS. *Journal of Pharmacognosy and Phytochemistry*.

[37] Ren J., Wang J., Karthikeyan S., Liu H., Cai J. (2019). Natural Anti-Phytopathogenic Fungi Compound Phenol, 2, 4-bis (1, 1-Dimethylethyl) From Pseudomonas Fluorescens TL-1. *Indian Journal of Biochemistry & Biophysics*.

[38] Padmavathi A. R., Abinaya B., Pandian S. K. (2014). Phenol, 2, 4-Bis (1, 1-Dimethylethyl) of Marine Bacterial Origin Inhibits Quorum Sensing Mediated Biofilm Formation in the Uropathogen *Serratia marcescens*. *Biofouling*.

[39] Lotfy M. M., Hassan H. M., Hetta M. H., El-gendy A. O., Mohammed R. (2018). Di- (2-Ethylhexyl) Phthalate, A Major Bioactive Metabolite With Antimicrobial and Cytotoxic Activity Isolated From River Nile Derived Fungus Aspergillus Awamori. *Beni-Suef University Journal of Basic and Applied Sciences*.

[40] Li C., He L., Jin G., Ma S., Wu W., Gai L. (2017). Effect of Different Irradiation Dose Treatment on the Lipid Oxidation, Instrumental Color and Volatiles of Fresh Pork and Their Changes During Storage. *Meat Science*.

[41] Arora S., Kumar G., Meena S. (2017). Gas Chromatography-Mass Spectroscopy Analysis of Root of an Economically Important Plant, *Cenchrus ciliaris* L. From Thar Desert, Rajasthan (India). *Asian Journal of Pharmaceutical and Clinical Research*.

[42] Hamouda A. F., Farawilla T.-L. M., Attafi I. M. (2021). Screening Pilot Study of Fruit Seed Compositions by GC-MS and Their Potential Scenario Anti ACE2 and 2rh1 Receptors as a Recycling Possibility in the Coronavirus Pandemic. *Journal of Clinical Medical Research*.

[43] Meenakshi V. K., Gomathy S., Senthamarai S., Paripooranaselvi M., Chamundeswari K. P. (2012). GC-MS Determination of the Bioactive Components of Microcosmus Exasperatus Heller, 1878. *TIC*.

[44] Fernandes E. S., Passos G. F., Medeiros R. (2007). Anti-Inflammatory Effects of Compounds Alpha-Humulene and (−)-Trans-Caryophyllene Isolated From the Essential Oil of Cordia Verbenacea. *European Journal of Pharmacology*.

[45] Mazumder K., Nabila A., Aktar A., Farahnaky A. (2020). Bioactive Variability and In Vitro and In Vivo Antioxidant Activity of Unprocessed and Processed Flour of Nine Cultivars of Australian Lupin Species: A Comprehensive Substantiation. *Antioxidants*.

[46] Nwanisobi G. C., Aghanwa C. I., Ezeagu C. U. (2021). Fatty Acid Composition of Ficus Sur Seed Oil (Moraceae) Obtained in Enugu State, Nigeria. *Journal of Chemical Society of Nigeria*.

[47] Krishnamoorthy K., Subramaniam P. (2014). Phytochemical Profiling of Leaf, Stem, and Tuber Parts of Solena Amplexicaulis (Lam.) Gandhi Using GC‐MS. *International Scholarly Research Notices*.

[48] Sachindra G. R., Vinay Kumar K. S., Eshan M. R. (2011). Cost and Management Accountants in Service Sector—Realistic Positioning of Cost and Management Accountants in Airline Industry. *Management Accountant-New Delhi*.

[49] Khan S., Kaur H., Jhamta R. (2019). Evaluation of Antioxidant Potential and Phytochemical Characterization Using GC-MS Analysis of Bioactive Compounds of *Achillea filipendulina* (L.) Leaves. *Journal of Pharmacognosy and Phytochemistry*.

[50] Petermann A. B., Reyna-Jeldes M., Ortega L., Coddou C., Yévenes G. E. (2022). Roles of the Unsaturated Fatty Acid Docosahexaenoic Acid in the Central Nervous System: Molecular and Cellular Insights. *International Journal of Molecular Sciences*.

[51] Crobeddu B., Ferraris E., Kolasa E., Plante I. (2019). Di (2-Ethylhexyl) Phthalate (DEHP) Increases Proliferation of Epithelial Breast Cancer Cells Through Progesterone Receptor Dysregulation. *Environmental Research*.

[52] Paudel M. R., Pant B. (2018). Cytotoxic Activity of Crude Extracts of Dendrobium Amoenum and Detection of Bioactive Compounds by GC-MS. *Botanica Orientalis: Journal of Plant Science*.

[53] Xiang F., Bai J., Tan X., Chen T., Yang W., He F. (2018). Antimicrobial Activities and Mechanism of the Essential Oil From Artemisia Argyi Levl. Et Van. Var. Argyi Cv. Qiai. *Industrial Crops and Products*.

[54] Silva F., Ferreira S., Queiroz J. A., Domingues F. C. (2011). Coriander (*Coriandrum sativum* L.) Essential Oil: Its Antibacterial Activity and Mode of Action Evaluated by Flow Cytometry. *Journal of Medical Microbiology*.

[55] Dsouza M. R., Bhat V. (2018). Evaluation of Pharmacological Activities of Seed and Pericarp of Litchi Chinensis Sonn. *Journal Homepage*.

[56] Bhat R. S., Al-daihan S. (2014). Antimicrobial Activity of Litchi Chinensis and Nephelium Lappaceum Aqueous Seed Extracts Against Some Pathogenic Bacterial Strains. *Journal of King Saud University Science*.

